# Of text and gene – using text mining methods to uncover hidden knowledge in toxicogenomics

**DOI:** 10.1186/s12918-014-0093-3

**Published:** 2014-08-13

**Authors:** Mikyung Lee, Zhichao Liu, Reagan Kelly, Weida Tong

**Affiliations:** 1 Division of Bioinformatics and Biostatistics, National Center for Toxicological Research, U.S. Food and Drug Administration, 3900 NCTR Road, Jefferson 72079, AR, USA

**Keywords:** Topic modeling, Toxicogenomics, Latent Dirichlet Allocation, Text-mining, Systems biology

## Abstract

**Background:**

Toxicogenomics studies often profile gene expression from assays involving multiple doses and time points. The dose- and time-dependent pattern is of great importance to assess toxicity but computational approaches are lacking to effectively utilize this characteristic in toxicity assessment. Topic modeling is a text mining approach, but may be used analogously in toxicogenomics due to the similar data structures between text and gene dysregulation.

**Results:**

Topic modeling was applied to a very large toxicogenomics dataset containing microarray gene expression data from >15,000 samples associated with 131 drugs tested in three different assay platforms (i.e., *in vitro* assay, *in vivo* repeated dose study and *in vivo* single dose experiment) with a design including multiple doses and time points. A set of “topics” which each consist of a set of genes was determined, by which the varying sensitivity of three assay systems was observed. We found that the drug-dependent effect was more pronounced in the two *in vivo* systems than the *in vitro* system, while the time-dependent effect was most strongly reflected in the *in vitro* system followed by the single dose study and lastly the repeated dose experiment. The dose-dependent effect was similar across three assay systems. Although the results indicated a challenge to extrapolate the *in vitro* results to the *in vivo* situation, we did notice that, for some drugs but not for all the drugs, the similarity in gene expression patterns was observed across all three assay systems, indicating a possibility of using *in vitro* systems with careful designs (such as the choice of dose and time point), to replace the *in vivo* testing strategy. Nonetheless, a potential to replace the repeated dose study by the single-dose short-term methodology was strongly implied.

**Conclusions:**

The study demonstrated that text mining methodologies such as topic modeling provide an alternative method compared to traditional means for data reduction in toxicogenomics, enhancing researchers’ capabilities to interpret biological information.

## Background

Toxicogenomics [1], or the application of genomic technologies to toxicology, has been recognized as having the potential to revolutionize toxicology. By measuring expression changes of tens of thousands of genes, we can identify mechanistic-relevant genes and pathways, improving our mechanistic understanding of toxicology. Nonetheless, toxicogenomics has fallen short of its initial promise [2]. While there is no single reason for this, one issue is that the current bioinformatics approaches used in toxicogenomics have not sufficiently dealt with the complexity of the toxicology study. For example, the assessment of a chemical’s toxicity requires data from experiments involving various doses and treatment durations and, in some studies, simultaneously applying several assay platforms. A single gene could have a dynamic profile across different treatment conditions (a combination of assay, dose and time point) with a role in multiple pathways which interact in complex manners to affect physiological changes of toxicity. Therefore, when analyzing toxicogenomics data, it is essential to ensure that this complexity is adequately captured.

The sheer scale of the data generated by toxicogenomics experiments prevents the easy identification of important genes. Instead, methods that cluster or group genes by their gene expression response and thereby reduce the dimensionality of the data are typically used. These include common statistical techniques such as hierarchical cluster analysis (HCA), principal components analysis (PCA) and *k*-means clustering. These tools have been widely applied to toxicogenomics data and other high-dimensional genomic data sources. However, a critical drawback to methods like HCA and *k*-means is the mutual exclusiveness of genes with respect to their involvement in biological processes (e.g., pathways) responding to exposure (i.e., HCA assigns one gene to one cluster that corresponds to a specific biological process, not to multiple clusters which actually is more relevant to the true event). Therefore, these methods often do not reflect the reality of the genomic response which limits our understanding of the complex interplay between genes and pathways. Exploring methods that are capable of holistically analyzing toxicogenomics data will improve the quality of the results and greatly contribute to mechanistic understandings of toxic response.

The genome is often referred to as a book of life: the genome has 30 billion letters (bases), ~25,000 words (genes) comprised by these letters, and many sentences/paragraphs (biological processes) that can be constructed with these words to associate with diseases that are repeated and spread across 23 chapters (chromosomes). Thus, one can conceptualize a relationship between genes and text, which share many commonalities and characteristics. For example, the same word can appear in different sentences while the same gene can be involved in different pathways. Such a commonality suggests that text mining tools could be useful alternative methods to analyze genomic data.

Topic modeling has been widely applied in the field of text mining, such as the mining of the enormous corpus of biomedical literature [3]. We applied this methodology to analyze FDA-approved drug labels for drug safety [4] and to explore drug repositioning opportunities [5]. Topic modeling considers a document to be a mixture of topics, and a topic to be a probability distribution over words. In many ways, a gene expression dataset resembles a set of documents; the dataset consists of mixtures of biological processes, which can be thought of as topics, and a biological process consists of a set of genes, which can be thought of as the words used to present a topic. In fact, topic modeling has already been successfully applied to the analysis of genome-wide biological profiling datasets. For example, Manuele et al. applied two different topic modeling approaches, PLSA (Probabilistic Latent Semantic Analysis) and LDA (Latent Dirichlet Allocation), for cancer classification using gene expression profiles [6]. Patrick et al. used a modified LDA technique to cluster drugs and genes [7]. Bing et al. applied a correspondence LDA model to discover microRNA regulated modules by identifying the microRNA and mRNA co-occurring frequently within the same latent variable [8].

While several examples mentioned above have successfully applied topic modeling to genomic datasets, the sizes of the studied datasets were small (less than 100). In addition, the utility of this method has not been explored in toxicogenomics in which the experiment design is usually complex (i.e., involving treatment at multiple-dose levels and different time points). In this study, topic modeling was applied to a large toxicogenomics dataset that contains gene expression data from over 15,000 samples [9]. The nature of the studied samples are heterogeneous and are generated from three different assay platforms but use the same set of 131 compounds, most of which are drugs. These contain data from an *in vitro* assay using rat primary hepatocytes, an *in vivo* assay in rats that employed a single dose treatment and an *in vivo* assay in rats that exposed them to repeated doses. The data were examined to determine how compounds and genes were grouped independently in terms of topics, or in this case, biological processes. These groupings were also extensively studied using network modeling and pathway analysis. In many places, “word” and “gene” as well as “document” and “treatment/experiment condition” were used interchangeably.

## Methods

### Dataset

The Japanese Toxicogenomics Project (TGP) is one of the most comprehensive efforts in the field of toxicogenomics, yielding a large dataset of gene expression profiles for 131 compounds, most of which are are drugs [9]. Specifically, its phase-I effort produced large-scale gene expression profiles for the effect of 131 compounds on rat livers using a short-term single-dose *in vivo* study (3, 6, 9 and 24 hours), a longer term study with multiple doses used repeatedly in *in vivo* experiments (4, 8, 15 and 29 days) and a study using multiple dose level *in vitro* experiments on rat primary hepatocytes (2, 8 and 24 hours). In total, 24 time/dose combinations for each of the 131 compounds were profiled for the *in vivo* samples while 9 time/dose combinations for each of the 131 compounds were profiled for the *in vitro* samples. Besides gene expression profiles, histopathological examination of the liver along with clinical chemistry and hematology data are also included in this dataset. Further information about this dataset, also known as TG-GATEs, can be found in Uehara et al. [9]. The dataset we used in this study was downloaded from CAMDA 2013 (http://dokuwiki.bioinf.jku.at/doku.php/start).

### Data processing

For each compound, gene expression profiles were generated for two control samples and three treated samples. As a preprocessing step, the probe-level data of the microarrays were quantile normalized followed with mapping of a probe set into corresponding genes [10], then multiple probes were summarized into one corresponding gene’s intensity ratio by using FARMS [11]. Next, we generated a “document” for each compound-assay-dose-time treatment condition, which contained “words” differentially expressed when compared with the matched control. A total of 12,088 genes were contained in the three assay systems (i.e. *in vitro*, *in vivo* single dose and *in vivo* repeat dose). We considered the same gene with a different transcriptional direction (i.e., up and down) as two different genes (just like a word and the same word with a prefix are two different words, such as boarding and pre-boarding), which led to a corpus of 24,176 words. The frequency of a word appearing in each document was determined by multiplying the fold change of the treated samples compared to the time-matched controls by 100 times. A total of 1,177, 1,564 and 1,563 documents representing a compound-dose-time combination were generated for the *in vitro*, *in vivo* single dose and *in vivo* repeat dose experiments, respectively.

### Topic modeling

LDA was applied to process the documents mentioned above [12]. LDA uses the Dirichlet prior probability to obtain topic distributions. The basic idea is that a document is represented as a mixture of several topics, where each topic is characterized by a word distribution. Thus, two Dirichlet distributions are employed, one for topic distribution over the documents and the other for word probabilities within a topic. These distributions are obtained by maximizing the posterior probability of observed documents. In this study, the open-source Mallet software package from the University of Massachusetts was applied. To determine the optimal number of topics to represent the dataset, we utilized the information loss and maximum likelihood approach to evaluate varying the number of topics ranging from 10 to 50. The modeling results include two different distribution files: topic distribution over document and word distribution over topic. The former includes the conditional probability of each topic given a document which, in this study, is a compound-assay-dose-time treatment condition, *P(T|D)*. This probability is a signature of the treatment, which will be used to assess similarity between samples assayed in different conditions. The latter represents the conditional probability of each gene (word) given a topic, *P(G|T)*, indicating which genes are important to a given topic.

### Clustering assays and compounds

After the LDA analysis, the conditional probability of each topic given a treatment condition, *P(T|D),* was obtained. For all possible sample (i.e., document) pairs from different treatment conditions, the Kullback–Leibler (K-L) divergence method, which is a measure of the difference between two probability distributions (P and Q, see the equation below), was applied to calculate similarities between any two samples based on conditional probabilities *P(T|D)*[13]:

(1) $$D\left(A,B\right)=\;\frac{D_{\mathrm{KL}}\left(A||B\right)+\;D_{\mathrm{KL}}\left(B||A\right)}{2}$$

(2) $$D_{\mathrm{KL}}\left(P||Q\right)=\;\sum_{i}P\left(i\right)\ln\frac{P\left(i\right)}{Q\left(i\right)}$$

where *i*, P(*i*) and Q(*i*) denotes the *i*th topic’s conditional probability given a document P and Q, respectively. Using the pairwise symmetrized K-L divergence defined in the equations (1,2), we identified the top 1% nearest document pairs. Then, those highly ranked document pairs were connected to each other in a network. In order to extract sub-networks, the MCODE plug-in for Cytoscape was applied to the constructed network which is designed to expand the cluster from highly interconnected seed nodes by setting a certain threshold [14].

### Functional analysis

The second result of LDA is the probability distribution of words within a given particular topic, *P(W|T)*. Specifically, *P(w*_*i*_*|t*_*i*_ *= j)* is the probability of gene *w*_*i*_ occurring in the *j*th topic, giving a measure of the importance of gene *w*_*i*_ to the *j*^*th*^ topic. Since topic modeling is designed to cluster words co-occurring frequently across whole documents, genes with a high rank in a topic are presumably involved in the biological processes determined by that topic. To determine the overrepresentation of biological processes for individual topics, functional analysis was applied with KEGG, and the significance test was based on Fisher’s exact test.

## Results

### Topic model development

The first step of applying topic modeling in toxicogenomics is to transform the gene expression measurements into a document-based format while retaining the information in the original dataset. A fold-change based transformation method was applied to convert the gene expression profiles of each compound-assay-dose-time treatment condition to a set of documents. Each document contained the genes that were dysregulated when comparing treated samples with the matched controls. The fold change value of a gene in a given treatment condition resembles a frequency of a word in a given document. Next, the number of topics optimally representing the information across all of the treatment conditions was determined using the information loss and maximum likelihood approach in a space of the number of topics between 10 and 50. After selecting 40 as the optimal number of topics representing this dataset, the topic model generated two probabilistic distributions. *P(T|D)* quantified the relevance of each topic (i.e., a conditional probability value) to a given treatment condition, thus a treatment condition can be characterized by the profile of 40 topics (the signature of a treatment condition). *P(W|T)* determined the importance of each gene (i.e., a conditional probability value) to a given topic. In this analysis, we used the top 300 genes with the largest *P(W|T)* value to represent each topic (Additional file 1: Table S1), resembling the meta-gene concept [15].

### Analysis of topics

**Figure 1:**
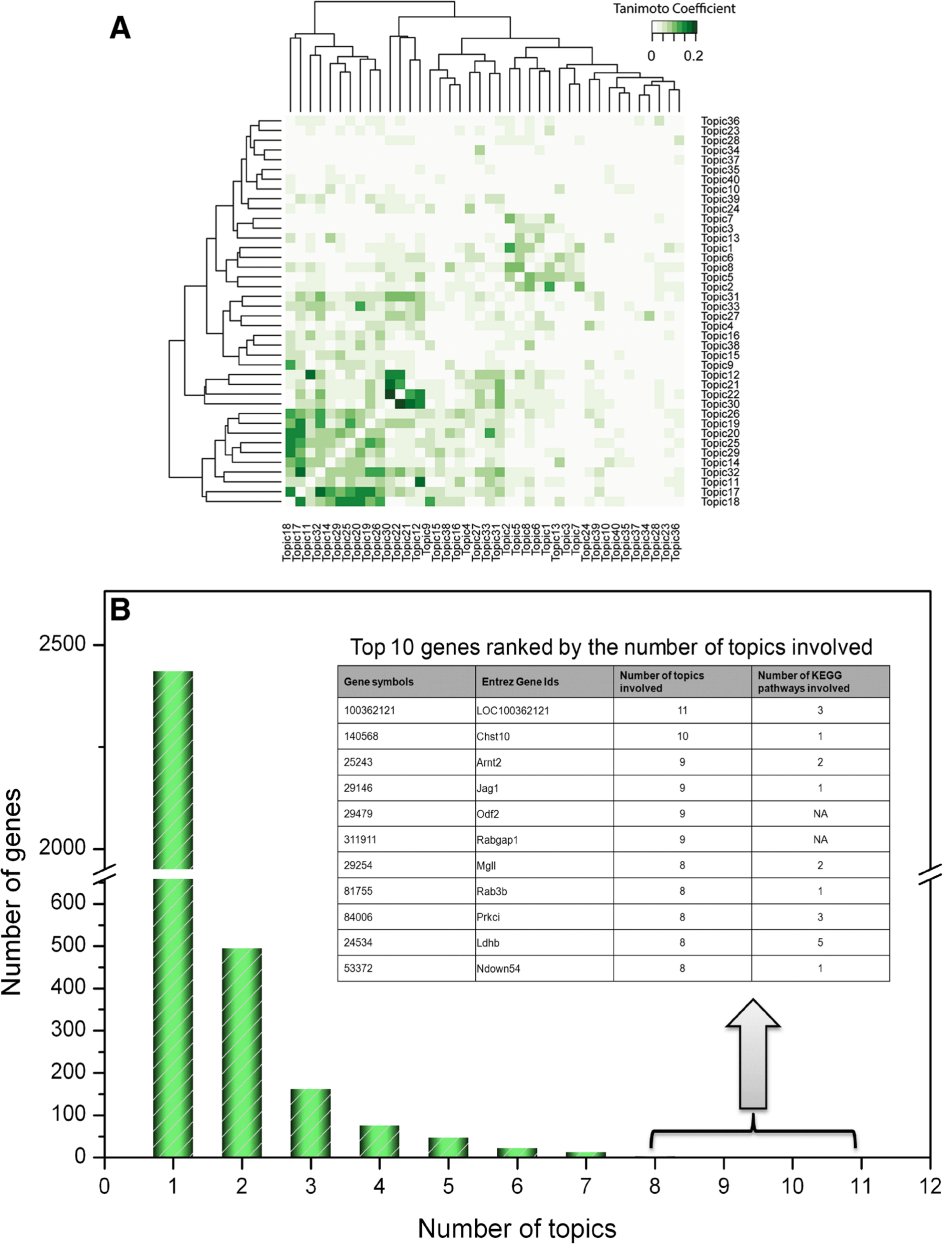
**Topic similarity and gene distribution over topics. (A)** The Tanimoto coefficient was calculated between topic pairs based on the top 300 genes. **(B)** Distribution of genes across all the topics.

Each of the 40 topics derived from the topic model was unique, as evident by a pairwise similarity assessment of topics using the Tanimoto method (Figure 1A) where the largest Tanimoto coefficient was only 0.2 for topics 22 and 30. The results implied that each topic represented a unique aspect of biology. Subsequently, the same genes presented in multiple topics could perform diverse roles leading to drug-induced toxicity in rat livers. As depicted in Figure 1B, the majority of genes only appear once amongst the 40 topics, while very few genes were presented in multiple topics. LOC100362121, also known as ddb2, appears in 11 topics. It is a damage-specific DNA binding protein, which involves various biological functions including protein autoubiquitination, protein polyubiquitination, pyrimidine dimer repair and participates in the nucleotide excision repair pathway. It interacts with 17β-estradiol [16], an endogenous estrogen that usually undergoes a substantial metabolic process in rat livers regulated by cychrome P450. It also interacts with TCDD (2,3,7,8-tetrachlorodibenzodioxine) and 2,4-D (2,4-Dichlorophenoxyacetic acid), two well-known environmental toxicants [17]. Additional file 2: Table S2 lists the genes appearing in more than 3 topics along with their annotation by MetaCore (https://portal.genego.com).

**Figure 2:**
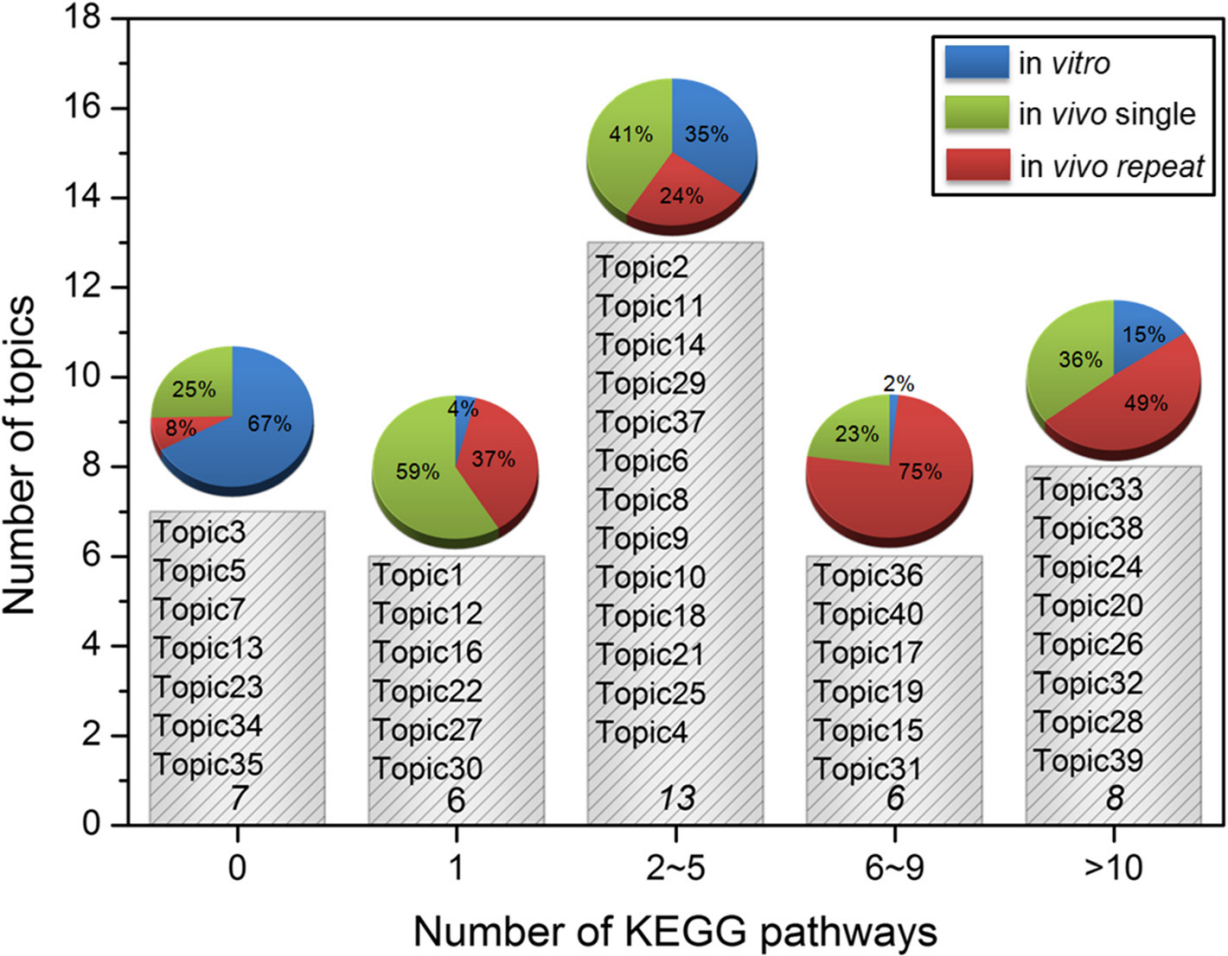
**Grouping topics by the number of significantly enriched KEGG pathways.** The pie chart above each bar shows the percentage of samples assayed in *in vitro* (blue), *in vivo* single dose treatment (green) and *in vivo* repeated dose treatment (red).

We also examined the over-represented pathways (P-value < 0.05 using Fisher’s exact tests) in each topic with KEGG and the results were summarized in Additional file 3: Table S3. A total of 199 pathways were identified, of which 70 pathways were uniquely represented. As shown in Figure 2, some topics had few over-represented pathways while others had many. We assigned each treatment condition to a topic with the largest conditional probability value of *P(T|D)* (Additional file 4: Table S4). We then examined how the choice of assay platform as well as dose level and treatment duration relates to the number of pathways over-expressed in different experiment conditions. As shown in the pie chart above each bar in Figure 2, the number of pathways elicited by three different assay systems followed a general order of *in vivo* repeated treatment > *in vivo* single dose test > *in vitro* assay. However, the trend is less clear at both dose and treatment time level.

### Assay’s sensitivity to the treatment effect by drug, dose or treatment duration

**Figure 3:**
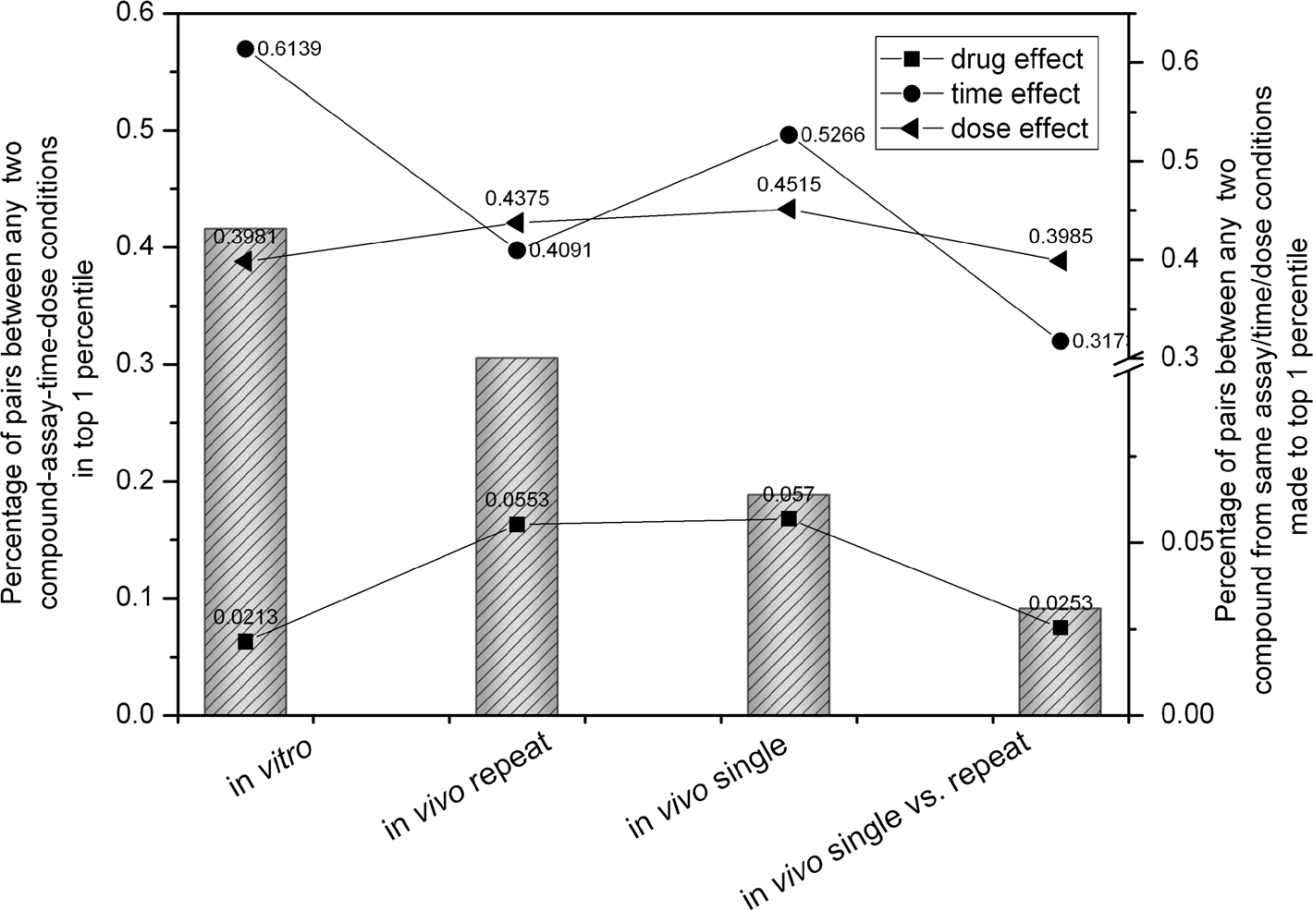
**Analysis of the top 1% nearest pairs (92,600 pairs) of any two compound-assay-dose-time experiment condition.** The bar chart (left axis) shows the percentage of pairs from individual assays in the top 1% pool while the line plot (right axis) assessed the treatment effect by drug, dose and time.

The most challenging aspect of this toxicogenomics dataset is that a single compound was often exposed to different doses and treated with different time durations in three experiment settings. These variables needed to be analyzed in an integrated fashion to determine the toxic potential of a compound. For that reason, we calculated the distance between any two treatment condition based on the probabilistic distribution of topics over the treatment condition (*P(T|D)*) using the K-L divergence, and a total of 9,257,900 pairs were generated. We selected 1% of the pairs (92,000 pairs) for analysis which had the highest pairwise similarity between two treatment conditions. A number of interesting observations were made (Figure 3).

Specifically, among the top 1% nearest pairs, 42% were from the *in vitro* method, followed by 30% from the *in vivo* repeated treatment and 19% from the *in vivo* single-dose experiment (bar chart of Figure 3). Similarly, the percentage of the pairs from each assay system within the top 1% nearest pairs also followed the same order of *in vitro* assay > *in vivo* repeated treatment > *in vivo* single-dose experiment (data not shown). Both findings strongly suggested that different assay systems have varying abilities to differentiate treatment conditions (compound-dose-time). Therefore, we investigated which assay systems are more sensitive to each treatment effect related to drug, dose or time. To assess the drug effect, we calculated the number of pairs for each bar (the pairs within the top 1% for each assay) in Figure 3 which had the same drug in a pair without considering dose and time. The same principle was used to estimate the dose and time effects. The results indicated that the drug effect was more pronounced in the two *in vivo* systems than the *in vitro* method (the bottom line in Figure 3). While the three testing methods had relatively similar sensitivity to the dose effect (middle line in Figure 3), the *in vitro* system clearly had a better sensitivity to the time effect followed by the *in vivo* single-dose method and the *in vivo* repeated dose approach (top line in Figure 3).

### Cross-assay extrapolation

Assessing whether the expensive *in vivo* repeated dose approach can be replaced by the short-term *in vivo* method or even an *in vitro* assay is of great interest to pharmaceutical industries and regulatory application. Therefore, in the top 1% nearest pairs, we also examined how many of them paired two different assays (the analysis did not consider the effect of compound, dose or time), an implication of a potential cross-assay extrapolation. As shown in the last bar of Figure 3, 9% in the top 1% pool paired two *in vivo* systems while none paired *in vitro* with any one of *in vivo* systems. The result suggested the potential use of a short-term assay with a single-dose treatment to supplement or replace the repeated dose study. This finding was further confirmed in a network analysis by connecting the top 1% nearest pairs followed with a clustering analysis using MCODE [14]. As depicted in Additional file 5: Figure S1, two large network clusters were formed along with many small ones, one associated with the *in vitro* assay alone and the other mixed both types of *in vivo* studies, implying that the *in vitro* system is sufficiently different from *in vivo* but two different types of *in vivo* assays share many commonalities at the transcriptional level.

**Figure 4:**
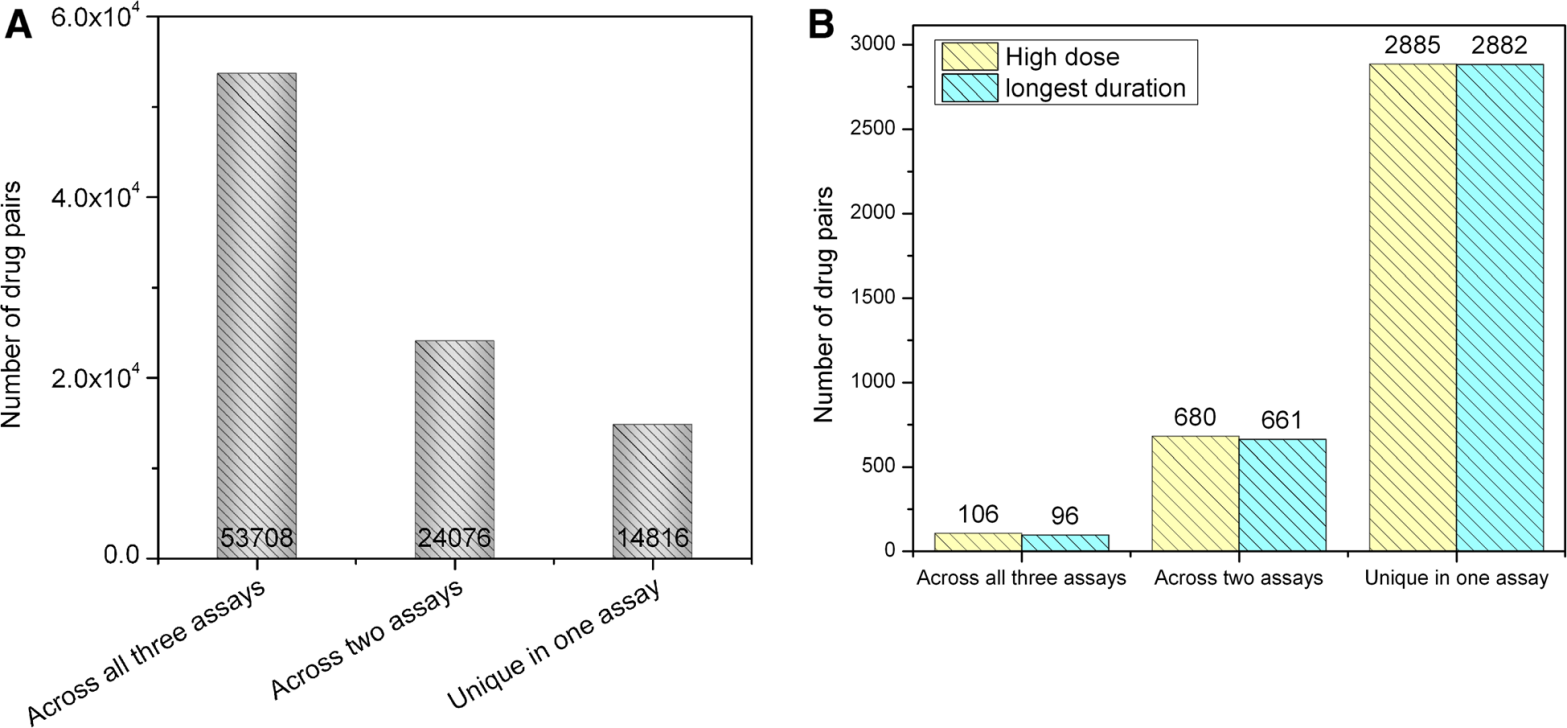
Percentage of drug pairs across any two or all three assays: (A) without considering dose and time point, and (B) applying the highest dose or the longest treatment duration.

Cross-assay extrapolation could be drug-dependent; some drugs may show a better consistency across assays than others. For that, we also examined the cross-assay extrapolation in the context of drugs. Specifically, in the top 1% nearest pairs, if we observed two pairs where pair 1 was between [*in vitro, drug A*, high dose, longest duration] and [*in vitro, drug B*, low dose, short duration] and pair 2 was between [*in vivo repeated dose, drug A*, medium dose, longest duration] and [*in vivo repeated dose, drug B*, high dose, longest duration], we considered that both the *in vitro* assay and *in vivo* repeated dose method had an equivalent ability to recognize the similarity between drug A and drug B. As shown in Figure 4A, more than half of these pairs were detected by all three assays without considering the effect of dose and treatment duration, followed with an additional 25% that were consistent in any two of three assay systems. The similar analysis was also conducted by using only high dose or longest treatment duration. As shown in Figure 4B, the drug pairs recognized by all three assay systems were significantly decreased, but a substantial number of drug pairs consistent across any two assays was observed. The results indicated that some drugs have a better extrapolation across assays than others.

### Network analysis

The network analysis mentioned in the previous section generated 108 subnetworks, ranging in size from 238 nodes to 3 nodes, with 25 nodes as the average network size (Additional file 6: Table S5). None of the subnetworks consisted of both *in vitro* and *in vivo* assays. A total of 28 subnetworks comprised of both *in vivo* single and repeated dose studies. Eight of the subnetworks contained nodes associated with a single compound, such as ethinylestradiol, ethionine, tamoxifen, colchicine and ethambutol with three of them in more than one assay system. For example, subnetwork 18 consisted of ten samples all treated with ethinylestradiol in the *in vivo* repeat dose study with different treatment conditions (i.e., 4-day medium and high dose, 8-day low, medium and high dose, 15-day medium and high dose, and 29-day low, medium and high dose treatment). Subnetwork 101 included three samples treated with ethinylestradiol in the *in vivo* single dose treatment. (i.e., 6-hour low dose and 9-hour low and medium dose). The findings are consistent with the histopathological changes seen with ethinylestradiol treatment (http://toxico.nibio.go.jp/); eosinophilic change is observed in almost all the time/dose points of the *in vivo* repeat dose assay. Tamoxifen, a synthetic estrogen sharing the similar mode of action with ethinylestradiol, has two subnetworks. One of them (subnetwork 40) includes six samples conditioned in the *in vivo* single dose study, and the other (subnetwork 79) includes four samples conditioned the *in vivo* repeated treatment. While some subnetworks mentioned above are enriched with a single drug using different time and dose conditions, some subnetworks (subnetwork 60, 83, 89) contained nodes associated with similar treatment conditions and assay types. For example, subnetwork 60 was composed of *in vitro* studies on four compounds (i.e., cyclophosphamide, simvastatin, tolbutamide, phenylanthranilic acid) that were each treated for 24 hours at the high dose level. It was found that three of these four drugs, all except phenylanthranilic acid (a chemical), are considered less likely to cause drug-induced liver injury (DILI) in humans as defined by NCTR's Liver Toxicity Knowledge Base (LTKB) [18] and two drugs, cyclophosphamide and simvastatin, belong to a same anatomical therapeutic category of cardiovascular system. Additionally, subnetwork 57 includes four drugs, clomipramine, danazol, nitrofurantoin and nitrofurantoin with 8 hours, medium or high dose condition in *in vitro* model, and all of them are most-DILI-concern defined by LTKB.

**Figure 5:**
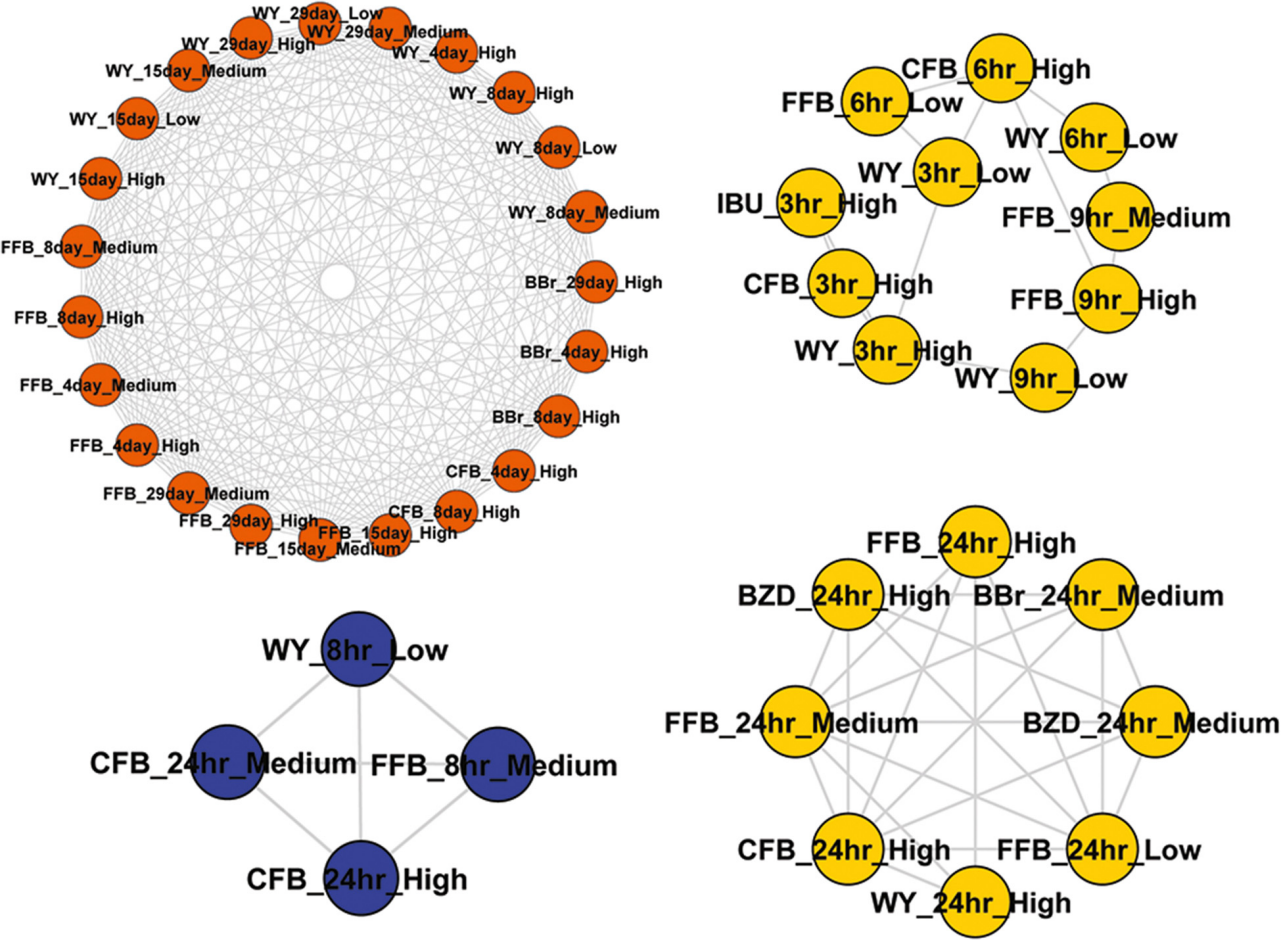
**Subnetworks 6, 26, 71 and 61 enriched with PPARα agonist.** Blue, yellow and orange nodes represent *in vitro*, *in vivo* single and *in vivo* repeat dose treatment, respectively. (WY: WY-14643, CFB: Clofibrate, FFB: Fenofibrate, BZD: Benziodarone, BBr: Benzbromarone, IBU: Ibuprofen).

We found that topic distributions can also be discriminative features for clustering of drugs by therapeutic category. Some drugs belonging to a certain anatomical therapeutic categories were clustered together such as cardiovascular system, Genito-urinary system and sex hormones, Musculo-skeletal system and Nervous system. Subnetwork 24 is composed of four distinct drugs in *in vivo* repeated treatment, clofibrate, gemfibrozil, simvastatin and benziodarone, all of which belong to the cardiovascular system. Subnetwork 16 is enriched with a therapeutic category of Musculo-skeletal system by including seven drugs, most of them are non-selective COX inhibitor. Interestingly, among a total of 12 samples, three of them (i.e., 4-day and 15-day on high dose treated with indomethacin, and 4-day on high dose treated with meloxicam) were conditioned on the *in vivo* repeated treatment while the rest of them (i.e., 24-hour on high dose treated with sulindac, 24-hour on high dose treated with diclofenac, 24-hour on medium and high dose treated with indomethacin, 24-hour on medium and high dose treated with lornoxicam, 24-hour on high treated with mefenamic acid and 24-hour on medium and high dose treated with naproxen) are from the *in vivo* single treatment, suggesting a potential replacement of the long-term assay with a short-term one. Intriguingly, PPARα agonists are clustered together in each assay system, as depicted in Figure 5. Subnetwork 6 includes 23 samples treated with three PPARα agonists (i.e., fenofibrate, clofibrate and WY-14643) in the *in vivo* repeated treatment while subnetwork 26 and 71 includes 8 and 10 samples in the *in vivo* single treatment with the same PPARα agonists along with the inclusion of two non PPARα agonists, ibuprofen and benziodarone. Both subnetwork 26 and 71 are enriched with the *in vivo* single study, although their treatment condition is different; subnetwork 26 is conditioned on longest duration (24 hour treatment) while subnetwork 71 is conditioned on 3, 6 and 9 hour. Benzbromarone (included in the subnetwork 6) is not a PPARα agonist, however it is known to have a high binding affinity for PPARα, showing potential as a PPARα agonist [19]. Ibuprofen (included in the subnetwork 71) is also known to be potential PPARα agonist [20]. Subnetwork 61 has four samples treated with three PPARα agonists, fenofibrate, clofibrate and WY-14643 in the *in vitro* assay. Subnetwork 43 is composed of three anti-cancer drugs, cisplatin, carboplatin and puromycin aminonucleoside. Both Cisplatin and carboplatin are platinum-containing anti-cancer drugs, cisplatin is a parent drug of carboplatin. Puromycin aminonucleoside is also an anti-cancer drug, inhibiting protein synthesis [21]. Subnetwork 63, enriched with a therapeutic category of Genito-urinary system and sex hormones, is composed of two drugs, danazol and methyltestosterone which act as androgen receptor agonist. Subnetwork 20 is composed of phenobarbital and acetaminophen in *in vitro* study which belongs to a nervous system. It is clear that topic modeling is capable of identifying biologically relevant topics in an unsupervised manner. It groups the drugs under the same therapeutic categories based on their associated topics, which may provide a new avenue for target identification and/or drug repositioning [5].

## Discussion

Two large toxicogenomics datasets were made publicly available recently: TG-GATEs and DrugMatrix [2]. While the availability of such large datasets generates tremendous opportunity, it creates challenges as well in the field of toxicogenomics. Both datasets apply a study design that includes multiple doses and treatment durations across different assay systems. The complexity of these datasets requires advanced data analysis methods to take advantage of dose- and time-dependent features in toxicity assessment. We explored the utility of topic modeling in toxicogenomics by analyzing the phase 1 of TG-GATEs dataset which includes data from >15,000 arrays derived from three different assay types (i.e., rat *in vitro* assay, rat *in vivo* single dose treatment and rat *in vivo* repeat treatment studies). By applying network analysis to the topic modeling results, we made several interesting observations about the impact of assay difference, dose and treatment duration.

Classifying samples based on gene expression profiling is a major focus in genomics research, including toxicogenomics. Most traditional clustering approaches (e.g., PCA, *k*-means and HCA) classify samples based on the gene-gene correlation principle. However, topic modeling considers samples as a mixture of latent topics and each topic is characterized by the probabilistic distribution of genes. This formula permits samples to be associated with multiple topics and genes to be associated with multiple topics. In doing so, each gene in topic modeling can be assigned to multiple topics, which is a key difference to the traditional unsupervised clustering methods where each gene is assigned to a single cluster. In this study, we focused our analysis on the top 1% similar pairs of treatment conditions based on topics. Subsequently, we generated the top 1% of similar pairs using the gene expression correlation matrix and compared it with the topic modeling results. The overlap between two approaches was only 21%, indicating that both methods capture different aspects of the biological process and could be complimentary each other to gain in-depth understanding of underlying mechanisms of toxicity.

Toxicogenomics usually applies an experimental design involving multiple time and dose points and different assay conditions. Such a design offers an opportunity to comprehensively address a number of key questions in toxicogenomics [2]. For instance, whether *in vitro* assays or short-term assays can supplement or even replace long-term *in vivo* assays, since the latter are much more time consuming and resource intensive. In this study, we observed similarities identified by the latent topic variable between two *in vivo* experiment designs (i.e., *in vivo* single dose vs. *in vivo* repeat dose treatment), indicating that the short-term *in vivo* assay with single dose treatment shares similar gene expression responses with the traditional repeated dose assay protocol. In contrast, distinct differences were observed between *in vitro* and *in vivo* responses.

The network analysis of the topic modeling results aims to cluster compounds in different treatment conditions with similar biological effects. Here, the pair-wise similarity between different treatment conditions (compound-assay-dose-time combinations) was generated based on topic distribution. This approach offers an alternative solution to study underlying toxic response. A network was developed using the top 1% most similar pairs of treatment conditions. The resulting network showed two distinct groups, one associated with the *in vitro* assay and the other for the *in vivo* assays. The network considers that two different *in vivo* assays (i.e., single-dose and repeated-dose) are similar, which is consistent with previous observations that a short-term *in vivo* experiment can offer comparable insight to long-term *in vivo* experiments [22]. The nodes were clustered according to similarity, generating a total of 108 subnetworks. Some subnetworks contained settings treated with varying amounts of the same compound. This suggests a response that is less sensitive to dosage. Some subnetworks, however, contained samples treated at similar dosage levels (e.g., high level) but with different compounds. These subnetworks are suggestive of compounds sharing similar mechanisms of action. A number of subnetworks were over-represented with a certain therapeutic category, non-steroidal anti-inflammatory drug, anti-cancer drug and PPARα agonist.

Together, our approach demonstrates that topic modeling offers several distinct benefits, particularly when applied to toxicogenomic expression profiling data. First, for high-throughput gene expression profiling, dimensionality reduction and visualization are key aspects in effectively analyzing and interpreting data. Topic modeling was able to reduce data dimension very effectively in terms of the latent variable, topic. Second, topic modeling is a soft clustering technique which does not assume mutual exclusivity and permits multiple topic assignment to the same sample and gene, reflecting true biological complexity. Third, the biological context associated with the topics can be easily interpreted by using functional analysis approaches such as GSEA [23].

## Conclusion

This study investigates the applicability of topic modeling for the clustering of gene expression profiles. Our results demonstrate that topic modeling offers an opportunity for use in the identification of hidden variables (topics) embedded in gene expression profiles. These topics can be discriminative features for clustering gene expression profiles. Additionally, the probabilistic representation of the topic model provides more flexibility for data interpretation. While the application of topic modeling methods to toxicogenomic data was the focus of this study, topic modeling can also be extended for analysis of similar data types such as data generated with next generation sequencing (NGS) methods.

## Competing interests

The authors declare that they have no competing interests.

## Authors’ contributions

ML performed all calculations and data analysis, and wrote the first draft of manuscript. WT, ZL and RK contributed to the data analysis, verified the calculations, and assisted with writing the manuscript. WT and ZL developed the original idea and guided the data analysis and presentation of results. All authors read and approved the final manuscript.

## Additional files

## Supplementary Material

Additional file 1: Table S1 Top 300 genes that represent each topic. Click here for file

Additional file 2: Table S2 Genes assigned to more than 3 topics with their functional role information. Click here for file

Additional file 3: Table S3 Functional analysis results for each topic: Fisher’s exact test with a p value cut-off of 0.05 was used in KEGG. Click here for file

Additional file 4: Table S4 Information for each topic, including Mode of Action (MoA), therapeutic category, and DILI annotation. Click here for file

Additional file 5: Table S5 Information about 108 subnetworks including Mode of Action (MoA), therapeutic category, and DILI annotation. Click here for file

Additional file 6: Figure S1 The global view of the network generated from the top 1% nearest pairs. Blue, yellow and orange represent the samples treated with a compound in *in vitro*, *in vivo* single and *in vivo* repeat, respectively. Click here for file
