# Supplementary figures and images for: Of text and gene – using text mining methods to uncover hidden knowledge in toxicogenomics
Source: BMC Syst Biol. 2014 Aug 13;8:93. doi: 10.1186/s12918-014-0093-3 (PMC4236689; doi:10.1186/s12918-014-0093-3)

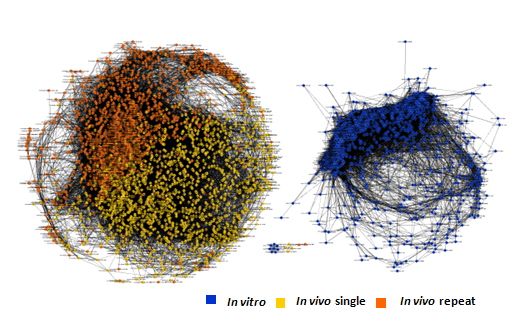

Supplement: Additional file 5: Table S5 — Information about 108 subnetworks including Mode of Action (MoA), therapeutic category, and DILI annotation. [file s12918-014-0093-3-S5.tiff]
